# An ecological and evolutionary perspective on the parallel invasion of two cross-compatible trees

**DOI:** 10.1093/aobpla/plw056

**Published:** 2016-08-12

**Authors:** Guillaume Besnard, Peter Cuneo

**Affiliations:** 1 CNRS, UPS, ENFA, Laboratoire Evolution & Diversité Biologique, UMR 5174, 31062 Toulouse 4, France; 2 The Australian PlantBank, Royal Botanic Gardens and Domain Trust, The Australian Botanic Garden, Mount Annan, NSW 2567, Australia

**Keywords:** Admixture, African olive, biotic interaction, ecological niche shift, introgression, invasive olive, *Olea europaea*, phylogeography, plant invasion management

## Abstract

Invasive trees are generally seen as ecosystem-transforming plants that can have significant impacts on native vegetation, and often require management and control. Understanding their history and biology is essential to guide actions of land managers. Here, we present a summary of recent research into the ecology, phylogeography and management of invasive olives, which are now established outside of their native range as high ecological impact invasive trees. The parallel invasion of European and African olive in different climatic zones of Australia provides an interesting case study of invasion, characterized by early genetic admixture between domesticated and wild taxa. Today, the impact of the invasive olives on native vegetation and ecosystem function is of conservation concern, with European olive a declared weed in areas of South Australia, and African olive a declared weed in New South Wales and Pacific islands. Population genetics was used to trace the origins and invasion of both subspecies in Australia, indicating that both olive subspecies have hybridized early after introduction. Research also indicates that African olive populations can establish from a low number of founder individuals even after successive bottlenecks. Modelling based on distributional data from the native and invasive range identified a shift of the realized ecological niche in the Australian invasive range for both olive subspecies, which was particularly marked for African olive. As highly successful and long-lived invaders, olives offer further opportunities to understand the genetic basis of invasion, and we propose that future research examines the history of introduction and admixture, the genetic basis of adaptability and the role of biotic interactions during invasion. Advances on these questions will ultimately improve predictions on the future olive expansion and provide a solid basis for better management of invasive populations.

## Abstract (precis)

The cultivated olive is an iconic Mediterranean crop that has been spread over the world in all regions with a Mediterranean climate. The species is however able to escape from cultivation, and can invade new ranges with negative impacts on native vegetation. The parallel invasion of two olive subspecies in different climatic zones of Australia provides an interesting case study of invasion, characterised by early genetic admixture between domesticated and wild taxa. In this synthesis, we provide an overview of the history and ecology of invasive olives, and identify further research needed to guide future management and invasion risk.

## Introduction

Exotic plant invasions are a major factor of global change and a significant threat to biodiversity (Mooney 2005). Increasing rates of plant introductions, linked to the expansion of global trade, suggest they will continue to pose conservation challenges in the future (Gallagher *et al.* 2010). In the recent past, the rate and risk associated with alien species introductions has increased enormously due to the rapid escalation in human alteration of the environment (Pimentel *et al.* 2001). Plant invaders can significantly alter the fire regime, nutrient cycling, hydrology and energy budget of native ecosystems (Mack *et al.* 2000). Through competitive interactions, they can directly reduce native plant diversity and abundance (Lake and Leishman 2004; Pyšek and Richardson 2006). To limit the impact of invasive species, it is essential that management actions are guided by knowledge of ecological requirements and evolutionary drivers favouring invasiveness.

Woody invaders are generally seen as ecosystem-transforming plants (Richardson and Rejmánek 2011; Nuňez and Dickie 2014). First, shading from invasive trees and shrubs has been repeatedly identified as detrimental to native understory diversity (e.g. Hobbs and Mooney 1986; McKinney and Goodell 2010). The conversion of vegetation from an open stand to a closed canopy will generally be accompanied by microclimatic changes such as higher humidity and lower temperatures (Gordon 1998). Second, woody plants have a high dependence on mutualists, both aboveground (for seed dispersal and/or pollination) and belowground organisms (e.g. mycorrhizal fungi; Nuňez and Dickie 2014). Consequently, tree invasions are generally associated with disruption of mutualistic interactions by species exclusion or recruitment of particular local species by facilitation (e.g. Mitchell *et al.* 2006). Tree invasions have also been linked to the co-introduction of soil microbes (e.g. Dickie *et al.* 2010; Ndlovu *et al.* 2013; Le Roux *et al.* 2016), potentially leading to major shifts in soil nutrient cycling that could in turn result in invasional meltdown (Dickie *et al.* 2014). Such ecosystem changes at both the macro- and micro-bial levels often make the restoration of invaded habitats a challenge for land managers (Traveset and Richardson 2011). The resilience of ecosystems is, however, variable and remains difficult to predict, and the role of mutualism and antagonistic interactions needs to be better documented (e.g. Palmer *et al.* 2008; Kaiser-Bunbury *et al.* 2010; Moeller *et al.* 2015).

Understanding the processes and factors leading to successful tree invasion has become a major topic in invasion biology (e.g. Richardson *et al.* 2007; Richardson and Rejmánek 2011; Zenni *et al.* accepted). Many trees and shrubs that have become invasive were introduced for specific purposes, for example forestry trees [e.g. *Acacia* spp. (Richardson *et al.* 2011; Thompson *et al.* 2015), *Eucalyptus* spp. (Doughty 2000), *Pinus* spp. (Richardson *et al.* 2007), *Prunus serotina* (Pairon *et al.* 2010)], ornamentals [*Ligustrum* spp. (e.g. Hoyos *et al.* 2010), *Miconia calvescens* (Le Roux *et al.* 2008)] or crops [e.g. Olive (Cuneo and Leishman 2006), *Psidium cattleianum* (Ellstrand *et al.* 2010)], while others have been accidentally introduced (e.g. Le Roux *et al.* 2008). Population genetic studies of invasive trees has revealed different histories of introduction, with invasion also strongly linked to the level of propagule pressure [e.g. usually high pressures in forest trees or crops, with multiple introductions from distinct provenances (Pairon *et al.* 2010; Le Roux *et al.* 2011; Mandák *et al.* 2013; Zenni *et al.* 2014) vs. low pressures in some ornamentals (Le Roux *et al.* 2008)]. Native populations of tree species often cover large areas and show considerable genetic variation in adaptive traits to fit local environmental conditions (e.g. seed size, seed dormancy, cold hardiness, bud phenology; Mátyás 1996; Savolainen *et al.* 2007). In the invasive range, genetic admixture between distinct provenances of trees has been documented (e.g. Le Roux *et al.* 2011; Zenni *et al.* 2014). This admixture may offer the possibility to produce new genotypic combinations (Ellstrand and Schierenbeck 2000), but natural selection remains the main driver in adaptive switches to a new range (Zenni *et al.* 2014). Importantly, phenotypic plasticity has been also reported to play a central role in the rapid evolution of invasiveness (e.g. Richards *et al.* 2012; Matesanz *et al.* 2012).

In this review, we examine the European olive (*Olea europaea* L. subsp. *europaea*), which is a major crop species and iconic tree of the Mediterranean region, and the related African olive (*O. europaea* subsp. *cuspidata*) which is a tropical wild olive primarily from southern and eastern Africa. Both these subspecies of *O. europaea* have become vertebrate dispersed invasive trees following horticultural introduction outside of their native range, particularly in Australia (Cuneo and Leishman 2006). As European and African olives originate from different climatic zones and are known to hybridize, they provide an interesting case study of parallel invasion with potential admixture in Australia that can be traced with molecular markers. We also provide an overview of the ecology of these invasive olives, and identify further research needed to guide future management and invasion risk.

## Geographic distribution and diversity of olives

Olives (*O.* *europaea* L., Oleaceae) are native to the Old World (Médail *et al.* 2001; Green 2002). Wild olives are naturally distributed over three continents in highly variable environments and thus considered to have high genetic diversity and adaptive capacity for naturalization and invasion in a wide range of habitats (Médail *et al.* 2001; Green 2002). Six olive subspecies are currently recognized but only two taxa have a large native distribution: the European olive (subsp. *europaea*) in the Mediterranean basin, and the African olive (subsp. *cuspidata*) from South-East Himalaya to Southern Africa. Molecular data have been used to investigate the diversification of the olive complex (e.g. Besnard *et al.* 2009). Several lineages have been described for olives based on various genetic markers, and their common ancestor dates back to the Late Miocene (Besnard *et al.* 2009). It is believed that the formation of the Saharan desert created a major geographic barrier to gene flow between North African-Mediterranean and Tropical African olives. Olive taxa are easily distinguished based on genetic data, but can hybridize when in contact leading to genetic admixture (e.g. Besnard and El Bakkali 2014; Cacères *et al.* 2015).

The cultivated olive tree originated from the Mediterranean Basin (Green 2002). It was probably first domesticated during the Copper Age in the Near East and underwent secondary diversification in central and western Mediterranean areas (Kaniewski *et al.* 2012; Zohary *et al.* 2012; Besnard *et al.* 2013). Since the beginning of historical times, the cultivated olive and wild relatives have been spread by humans for various reasons (e.g. olive production, rootstocks, ornament or forage; Carrión *et al.* 2010; Margaritis 2013; Besnard and Rubio de Casas 2016). In the native range, population turnover of olives is considered to be slow since millennial wild or cultivated trees are known in different places (e.g. Baali-Cherif and Besnard 2005; Arnan *et al.* 2012; Bernabei 2015). In addition, when the tree is cut or destroyed aboveground, for instance through fire or heavy frost, even ancient trees are able to resprout (e.g. Baali-Cherif and Besnard 2005; Therios 2009). This strategy allows for long persistence of olive individuals, and may partly explain the symbolism associated to this species (Kaniewski *et al.* 2012).

**Figure 1.**
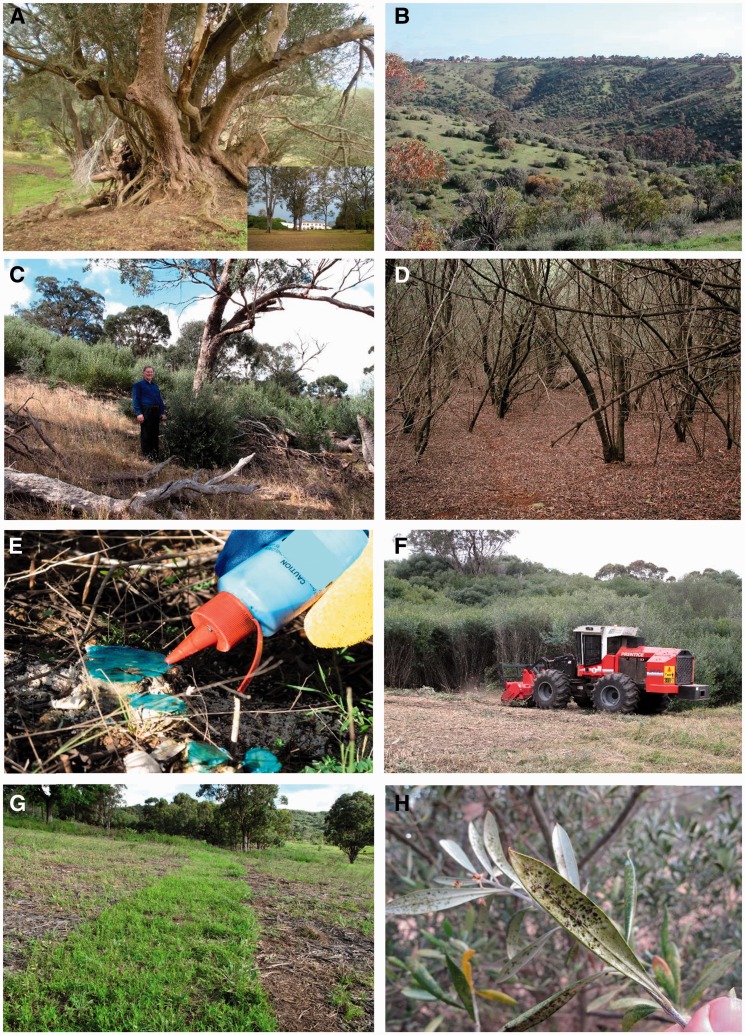
Illustrations of invasive olives in Australia. (A) Centennial olive tree (identified with genetic markers as a hybrid between European and African olives; Besnard *et al.* 2014) in the Camden Park Estate, NSW. This property (detail at the bottom right) is well known for the horticultural introduction of numerous plants into Australia during the 19th century; (B) European olive invasion in Adelaide Hills, SA. Abandoned pastures are rapidly colonized; (C) European olive ‘halo’ around the base of native eucalypt due to bird dispersal of seed; (D) low-plant diversity beneath dense African olive canopy (Mount Annan, NSW); (E) ‘Cut and paint’ herbicide application technique. Plants are cut as close as possible to ground level and undiluted glyphosate is quickly applied to the stump. Other methods can be used in areas where physical olive cutting is not practical such as the ‘Drill and Inject’ technique or the application of a broadleaf selective herbicide that leaves grasses intact to provide competition against weed re-establishment (Blood 2002); (F) mechanical control of African olive using ‘forest mowing’ technique (Mount Annan, NSW). This approach is used extensively in the arboriculture and vegetation management industry, and commonly utilizes earthmoving equipment. This type of machinery is able to mulch and process all above ground vegetation *in situ*, which is retained on site as mulch to prevent erosion and provide a level of weed suppression. Olive stumps are retained *in situ* to ensure soil stability, with stumps > 30 mm spot treated with herbicide and diesel; (G) field grass germination after direct sowing on cleared olive site; (H) olive lace bug (*Frogattia olivinia*), an Australian native pest of olives. This insect is a sap-sucking insect which feeds on the underside of the leaf, causing a yellow mottling of the leaf surface.

Since the 19th century, European and African subspecies have been introduced and become invasive in South East Australia and New Zealand, but the African olive has also established as invasive in distant tropical oceanic islands (e.g. Hawaii, Norfolk, Kermadec, Saint Helena; Cuneo and Leishman 2006; GISD 2010). The European olive tree was one of the earliest plants introduced to Australia by gardener George Suttor in 1800 and agricultural pioneer John Macarthur in 1805, but multiple new clones (varieties) have since followed (Sweeney and Davies 1998; El Kholy *et al.* 2012). The African olive was also brought into Australia by the Macarthur family, and is listed in the 1843 Nursery catalogue of Camden Park (Campbelltown, NSW; Fig. 1A). The reasons for the initial introduction of this wild taxon are unclear, but its uses as rootstocks for the cultivated olive and as a hedging plant are believed to be the main purposes.

**Figure 2.**
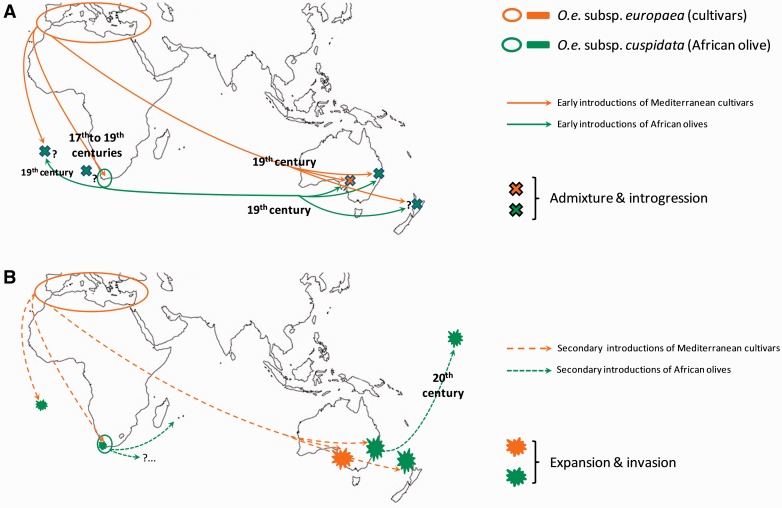
Scenario of an invasion story. (A) Early human-mediated spreads of European cultivated olives (*Olea europaea* subsp. *europaea*) and wild African olives (*Olea europaea* subsp. *cuspidata*) before the end of the 19th century. The quasi-simultaneous introduction of cultivars and African olives in Australia (between the 1800s and 1840s) was followed by early admixture in SA and NSW, as testified by genetic analyses (Besnard *et al.* 2007, 2014). The same phenomenon is likely to have occurred in St Helena, Northern New Zealand and Norfolk Island, where olives have also been early introduced (Cuneo and Leishman 2006; Lambdon 2012). Additionally, admixture between European and African olives could have as well occurred in the native range where cultivars have been introduced in the Cape area early after the European settlements (Wilmot 1869). These hypotheses (indicated by question marks) still need to be confirmed with genetic analyses. It is also not yet excluded that independent introductions from the Cape area to Australia, New Zealand and St Helena have occurred, and additional genetic analyses based on nuclear markers are necessary to test these hypotheses; (B) after a lag time (i.e. selection of new gene combinations more adapted to local conditions) olives have spread in the landscape and mainly colonized anthropogenic habitats (e.g. abandoned pastures). Such African olive expansion was also observed within the native range, in Western Cape (e.g. Stellenbosch countryside, G. Besnard, personal observation). In parallel, new European olive cultivars were still introduced in the same areas increasing the probability of admixture with invasive olives. Further secondary introductions of African olive from the invasive range (NSW) to new ranges (e.g. Hawaii) were demonstrated with genetic analyses (Besnard *et al.* 2014). Additional recent translocations from South Africa to new areas are poorly documented but likely to have occurred (e.g. trade of ornamental trees at La Réunion). The depauperate nuclear genetic diversity in African olives from Australia and Hawaii as well as the occurrence of the same two African plastid haplotypes in the whole invasive range (i.e. East Australia, Norfolk, Kermadec, North New Zealand, Hawaii and St Helena) suggest a spread from a limited number of founders from the Cape area (Besnard *et al.* 2014).

A phylogeographic approach has been used to identify the origins of invasive olive populations in Australia and other locations (Fig. 2; Besnard *et al.* 2007 2014). Polymorphisms from maternally (plastid DNA) and biparentally inherited (nuclear DNA) genomes revealed that European olive populations from South Australia (SA) mostly originated from the Mediterranean Basin, and derived from multiple cultivar introductions (Besnard *et al.* 2007). In contrast, African olive populations from NSW, Norfolk Island, northern New Zealand, Hawaii and Saint Helena mainly originated from South Africa, harbouring two plastid haplotypes detected in the Western Cape (Besnard *et al.* 2014). African olive seed or tree introduction from South Africa was certainly from an area around Cape Town controlled by Europeans during the early 19th century (Wilmot 1869) and accessible for collecting propagules. For example, African olive is abundant in the Kirstenbosch National Botanical Garden (which was established during the 17th century), and is frequently seen in the surrounding countryside where it can colonize anthropogenic habitats (G. Besnard, personal observation). Nuclear genes also indicated that hybridization between the two introduced olive subspecies has occurred in Australia, both in SA and NSW (Fig. 2A). This hybridization was probably early after the introduction of olives, since a high density of hybrids (all the 26 analysed individuals) was detected in the historic property of Camden Park, well known for the horticultural introduction of plants to Australia during the 19th century (Besnard *et al.* 2014).

Multiple variety introductions from different origins have maintained a relatively high overall diversity in the Australian cultivated European olive germplasm (Sweeney and Davies 1998). As a consequence, relatively high gene diversity was detected in olive populations from South Australia (Besnard *et al.* 2007, 2014). In contrast, successive bottlenecks occurred during the invasive history of the African olive, first from South Africa to Australia, and then from Australia to oceanic islands (Besnard *et al.* 2014; Fig. 2B). Testing population demography scenarios suggested that the initial effective population in Maui (Hawaii) was very small, likely with less than ten individuals (median *N*_e_  =  9.8; Besnard *et al.* 2014). As a consequence, the African olive gene pool on Hawaii is particularly depauperate.

## Biology and ecological impact of invasive olives: lessons from Australia

*Seed and pollen dispersal*: Efficient gene dispersal mediated by pollen and fruits should favour colonization of suitable habitats and maintain connectivity between distant patches (e.g. Sork 2015). In olives, while pollen is mainly wind-dispersed, fleshy fruits are rich in oil and particularly attractive to frugivores (e.g. birds, rodents) that ensure their natural dispersal. In the invasive range, fruit size and avian dispersal are key factors driving the spread of olives. Fruit dispersal has been reported over tens of km (Aslan and Rejmánek 2011). Birds are less able to manipulate and swallow fruits wider than 11.83-mm diameter (Altcántara and Rey 2003); however, the normal fruit size of ∼7 mm for African olive is optimal for dispersal by native and introduced birds (Cuneo and Leishman 2006). When cultivated olive groves of European olive are abandoned, the fruit of self-seeded olive trees are smaller than the original cultivars, and avian fruit dispersal may thus increase (Spennemann and Allen 2000).

*Growth and initial establishment*: Although multiple factors are involved in the success of an invasive tree species, growth rate has been identified as a good predictor of invasiveness (Lamarque *et al.* 2011). On one hand, there are no known studies comparing the growth rates of *O. europaea* in native and invasive range, however, observations indicate that growth rates are similar between native and invasive locations where the climatic conditions are similar (P. Cuneo and G. Besnard, personal observation). On the other hand, the capacity to produce profuse dense seedling ‘mats’ beneath the canopy of established trees is a remarkable feature of the African olive in its invasive range (Cuneo and Leishman 2006). Indeed, African olive seedling densities of 950 seedlings/m^2^ are commonly observed in Australia, a regenerative capacity never observed in the native range (Cuneo and Leishman 2006). Such a high establishment capacity has also been reported in other invasive trees, in particular in privets (*Ligustrum* spp.; Hoyos *et al.* 2010; Greene and Blossey 2012).

*Impact of olives in Australia*: European olives are now well established as invasive in the Adelaide hills, SA, where the climate is comparable with the Mediterranean region (Fig. 1B and C). The majority of invasive stands occur in areas of former woodland with fertile, slightly acidic soils and 400–600 mm rainfall. The invasive populations harbour diseases and pests such as the olive fly that can cause crop losses in managed olive groves, but their key ecological impacts are the displacement of native vegetation and increased fire risk (DWLBC 2005). European olive reduces the abundance and diversity of native plant species, altering the canopy structure of the woodland and preventing native regeneration. Native canopy cover may be reduced by up to 80 % and native species diversity up to 50 % (Crossman 2002).

The presence of the invasive African olive has similar ecological impacts in NSW. A mapping study showed clearly that African olive is widespread and well established as invasive across a large region of western Sydney (Cuneo *et al.* 2009). In Cumberland Plain Woodland (South-West Sydney, NSW), where the native vegetation cover has been reduced to 13 % of its original extent (DECCW 2010), field surveys and a manipulative shading experiment showed that light levels under African olive cover were substantially reduced compared to native woodlands (canopy openness of 4 % and 50 %, respectively), and there were 78 % fewer native species under African olive (Fig. 1D) compared with un-invaded woodland sites (Cuneo and Leishman 2013). African olive was able to maintain an 88 % survival rate under a dense olive canopy (Cuneo and Leishman 2013). A study of invertebrate species richness for successive stages of African olive invasion (Nguyen *et al.* 2016) also revealed that diversity was significantly reduced under mature African olive stands compared to early-stage olive and mature native woodland. These studies confirmed the adaptability of African olive and its ability to act as an ‘ecosystem transformer’ by decreasing native plant and invertebrate diversity and substantially modifying native communities.

The ecological impact of invasion by both olive subspecies on native plant diversity is relatively high compared with the impact of other woody invasives. For example, *Ligustrum sinense* (Chinese privet) invasion resulted in a 41 % decrease in native plant diversity (Merriam and Feil 2002), and *Acer platanoides* (Norway maple) invading native *Fagus grandifolia* forest resulted in 36 % fewer native species beneath the shade of the Norway maple canopy (Wyckoff and Webb 1996). A study of *Acacia saligna* invasion of native fynbos vegetation in South Africa was also shown to reduce native plant diversity by 63 % at three long-term invaded sites (Holmes and Cowling 1997). Considering their negative impacts on native ecological communities in Australia, olives are now recognized by state agencies as a significant threat to the remnant vegetation, in particular the African olive in the Cumberland Plain and the Hunter Valley, north of Sydney. There are 12 ecological communities of the Cumberland Plain listed as endangered under state and federal legislation (DECCW 2010), justifying management actions to reduce the impact of the African olive.

## Management and control of invasive olives in NSW

Both European and African olives are considered by land managers and bush regeneration practitioners as persistent woody weeds difficult and expensive to control (West 2002; Crossman *et al.* 2003; DWLBC 2005). In the case of African olives, maintenance of a ‘natural’ fire regime (5–10 year recurrence interval) or the use of fire as a management tool has not proven effective in controlling the introduced in favour of native species, as established African olive (> 20 mm stem diameter) individuals are able to resprout from a lignotuber after fire in a manner similar to native species such as Eucalypts and Banksias (von Richter *et al.* 2005).

Control of olives invading native vegetation is best achieved at the incipient stage, particularly as olive seedlings first appear as ‘halos’ beneath large perch trees and there are components of the native understory layer still present (Fig. 1C). In contrast, the control of dense monoculture African olive forests (Fig. 1D) that are widespread throughout South-West Sydney (NSW) is a critical issue. Control of this advanced ‘forest’ stage of African olive invasion is expensive, and is currently achieved through a combination of herbicide application and mechanical land clearing (Fig. 1F; Cuneo and Leishman 2015). Cleared olive sites are then monitored for re-sprouting stumps and germinating seeds.

Cuneo *et al.* (2010) showed that the persistence of African olive seed in soil was ∼2.4 years. Its seed viability thus declines rapidly and provides a narrow window of opportunity for germination and regeneration. Persistence in the soil seed bank is indeed short compared with other invasive species, particularly hard-seeded legumes such as Broom (*Cytisus scoparius*) which forms persistent soil seed banks (>5 years; Thompson *et al.* 1993). This also means that once mature African olive trees are removed, control of seedlings germinating from the seed bank should be required only for 2–3 years, along with monitoring of seedlings derived from dispersal by birds into the managed site. In contrast, European olive seeds differ from those of the African olive, as it has a thicker woody endocarp and physiological dormancy of the embryo that leads to slow germination under horticultural and field conditions. This dormancy of the embryo is present even when the endocarp is removed (Rinaldi 2000), and seeds can retain high germinability after storage for three years (Fabbri *et al.* 2004). Although there are no studies of European olive persistence in the soil seedbank available, the combination of resistant endocarp and dormancy suggests longer seed persistence than for the African olive.

Removal of invasive olives using the methods described above will not be sufficient to restore the original native eucalypt woodland vegetation, particularly for degraded sites where dense olive stands have developed over several decades. Active restoration and promotion of native regeneration will be required as part of a strategy to promote native plant diversity, control weeds and achieve sustainable woodland landscapes (Prober and Thiele 2005). The combination of short soil seed bank persistence in the African olive combined with relatively unaltered soil chemistry after long-term African olive invasion (Cuneo and Leishman 2015) provides an opportunity to restore these degraded sites through direct seeding of native species and stimulation of the native soil seed bank. Experimental work by Cuneo and Leishman (2015) showed that native grasses were absent from the soil seed bank in highly degraded African olive sites but direct seeding was able to re-establish a native perennial grass cover (Fig. 1G), which was resistant to subsequent weed invasion. This grass cover could be managed as an important first stage in woodland restoration, with exotic broadleaf species controlled by fire and/or selective herbicide. The resilience of native species was evident in the fire-stimulated germination of several hard seeded native species from the soil seed bank after 15 years of African olive invasion (Cuneo and Leishman 2015). The results of this restoration experiment were used to develop a ‘bottom-up’ model of ecological restoration, where restoration efforts focus initially on the establishment of a dense perennial grass cover as an early successional stage. Fire can be used in subsequent years to provide interstitial gaps for further direct seeding, and additional stimulation of soil seed bank germination.

## Ecological modelling and predictions of future invasive dynamics

**Figure 3.**
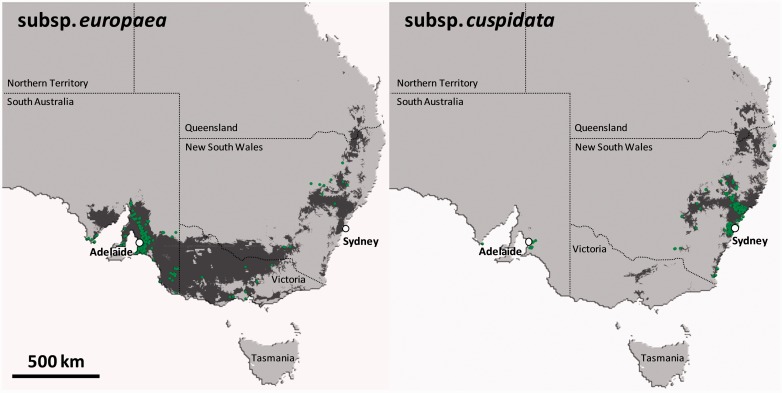
Distribution of both olive subspecies (subspp. *europaea* and *cuspidata*) in the Australian range as predicted by Species Distribution Models calibrated with data from the invaded range (based on observed occurrences indicated by small green points). The GARP software (Stockwell and Peters 1999) was used for the niche modelling analyses based on 37 environmental variables. These consisted of 19 climatic variables (including annual trends, seasonality and extreme climatic parameters measured over the last 30 years), 10 variables of soil properties, 7 land cover variables and altitude [see Cornuault *et al.* (2015) for detailed analyses]. Although the infestation presently occurs around Adelaide (subsp. *europaea*) and Sydney (subsp. *cuspidata*), models predict a potential spread over large areas in south-eastern Australia. Note that the predicted distribution is not continuous with a large gap between NSW and SA/Victoria states, but potentially suitable habitats for both subspecies largely overlap in NSW.

There are a number of bioclimatic modelling studies (based on climatic, soil and land cover variables) which are relevant to the potential olive distribution under current and future climates in Australia (Crossman and Bass 2008; O’Donnell *et al.* 2012; Cornuault *et al.* 2015; Roger *et al.* 2015). The parallel invasion of European and African olives was recently investigated in south-eastern Australia (Cornuault *et al.* 2015; Fig. 3). By comparing the ecological requirements of native and invasive olives, it was shown that the spatial segregation of the two subspecies in their non-native range was partly determined by differences in their native niches (i.e. niche filtering; Cornuault *et al.* 2015). However, a realized niche shift occurred through a contraction of the native niche in both subspecies. Although niche shifts are considered to be rare in invasive plants (Petitpierre *et al.* 2012), such changes were already highlighted by Gallagher *et al.* (2010) on 20 species exotic to Australia and also in the invasive range of *Pinus taeda* by Zenni *et al.* (2014). The reason for these rapid shifts is not yet identified. The adaptation and expansion of olives into these new habitats could be due to the selection of new gene combinations and/or the high level of observed phenotyic plasticity, combined with an absence of some stresses in a new range (see below).

It was also shown that olives have not yet colonized their full potential distribution area in Australia based on current conditions (e.g. O’Donnell *et al.* 2012; Cornuault *et al.* 2015; Roger *et al.* 2015). Cornuault *et al.* (2015) predicted that suitable habitat for the European olive covers a large region from Adelaide to Melbourne, expanding further north into the plains west of Sydney and Brisbane (Fig. 3). In contrast, the invasion of African olive invasion should be concentrated in eastern NSW and south-eastern Queensland. According to Cornuault *et al.* (2015), African and European olives share suitable habitats in NSW, Queensland and north of Melbourne (Fig. 3). Modelling presented in O’Donnell *et al.* (2012) and other studies on invasive species under future climate (Scott *et al.* 2008; Kriticos *et al.* 2010; Roger *et al.* 2015) predict a pattern of coastal and southerly retreat for temperate exotic plant species as a result of a warmer and drier climate. Future predictions of range contraction in invasive *O. europaea* have to be taken with caution, however, since the assumption of niche stasis may be unreasonable (Cornuault *et al.* 2015), especially with admixture between the two subspecies. In addition, it is increasingly evident that the response of invasive species to future climate change is likely to be strongly species- and context-dependent (Leishman and Gallagher 2015).

## Future research directions

As presented in this review, olives are becoming increasingly naturalized and invasive, and are now considered to be ‘next generation’ invasive trees. The combination of abundant seed crops/propagule pressure and vertebrate seed dispersal are key factors in their establishment and spread, but other biotic and abiotic factors could also be involved. Invasive olives provide an excellent case study of parallel invasion of two closely related taxa with considerable research opportunities, particularly based on the genetic data now available. The key future research questions that target the success of invasive olives are presented in Table 1, and include; history of introduction with possible admixture between distant provenances, the genetic basis of their adaptability during invasion, the role of biotic interactions (e.g. with belowground native or co-introduced mutualists) and options for bio-control. Advances on these questions outlined below will ultimately improve our predictions on future expansion, and provide a solid basis for better management of invasive olive populations.

**Table 1.** Summary of main topics on olive invaders and proposed methodologies to address specific questions.

Topic	Specific questions to address	Potential approaches
History of the invader	- Non-independent origins of invasive populations (e.g. African olive in Australia, Hawaii, New Zealand, Norfolk, St Helena) - Importance of admixture and introgression - Past population dynamics (e.g. initial population size, number of generations)	- Phylogeography and population genetics - Genomic scan analyses - Demography scenario testing (e.g. Approximate Bayesian Computation)
Reasons for ecological niche shift	- Adaptation to new habitats - Identification of genetic and/or non genetic factors (impact of domestication?)	- Common garden experiments - Association mapping and landscape genomics
Role of biotic interactions	- Co-invasions (e.g. mycorrhiza, bacteria) - Novel interactions in the invasive range (e.g. microbiome, fauna, plants)	- Ecological field studies - Study of the invader-associated and local microbiome (omics)
Monitoring of invaded areas	- Predicting future invasion - Defining control strategies and assessing impact and success of restoration practices	- Ecological modelling - Field experiments

*History of the invaders*: While the great lines of the invasive olives’ history have been studied with phylogeographic and population genetic approaches (Besnard *et al.* 2007, 2014; Besnard and El Bakkali 2014), some important questions remain to be addressed on this matter (Fig. 2).

First, the origins of some invasive populations of African olive have not yet been properly investigated. In particular, we know that invasive populations from Saint Helena share a common maternal origin (from Western Cape) with those of Australia, New Zealand and Hawaii (Besnard *et al.* 2014), but it is unclear whether these introductions are independent or sequential. This could be tested with population genetics (e.g. Estoup and Guillemaud 2010). Identifying introductions as independent or sequential could be essential to interpret patterns of invasion (e.g. multiple evolution of invasiveness vs. expansion from a common cradle).

Second, the earliest introductions of cultivated European olive in the Western Cape are potentially ancient (i.e. following early stable European settlements in the Cape area at the 17th century; Wilmot 1869) and occurred before the introduction of the African olive to Australia. A first contact between African and European olives could have thus happened in the native range (as reported in *Acacia pycnantha*; le Roux *et al*. 2013). It seems likely that hybridization between these two subspecies has taken place in South Africa, because the African olive is often present in anthropogenically disturbed habitats in close contact with cultivated olive groves (as in NSW). The importance and scale of this phenomenon is, however, unclear. It could thus be relevant to compare patterns of genetic admixture and recent population dynamics of olives in the invasive range (Australia, New Zealand) and in natural and anthropogenically disturbed habitats of the Cape area. Describing the genome structure of both native and invasive trees will allow testing the hypothesis of introgression (i.e. incorporation of a gene or small genomic blocks from one entity into the gene pool of a second, divergent entity) from one subspecies to another during the early steps of invasion. Comparative genomics would also allow for testing the role and relative importance of inter-taxa recombination, and whether this has the potential to increase evolutionary changes and produce phenotypes that are better suited to colonize novel environments (e.g. Ellstrand and Schierenbeck 2000; Facon *et al.* 2006; Lavergne and Molofsky 2007; Zenni *et al.* 2014).

*Prediction of areas at risk for future invasion and causes for the rapid adaptation to new habitats*: Considering the need to limit the expansion of the olives in Australia, it is now essential that existing populations are mapped accurately, and any new incursions in areas identified as being bioclimatically suitable are closely monitored or controlled at an early stage. Refined models to confidently predict olive distribution should be very useful to better identify areas at risk for future invasion. In particular, potential niche shifts have to be considered carefully in such models (see above). Feedbacks with native and co-introduced biota are poorly known and not integrated in most predictive models (Guisan *et al.* 2014). It is also essential to identify the drivers behind the realized niche shifts reported by Cornuault *et al.* (2015). Non-genetic or genetic factors could be involved. Indeed, the olive is able to modulate expression of phenotypes (i.e. plasticity) according to surrounding conditions (e.g. García-Verdugo *et al.* 2009; Rubio de Casas *et al.* 2011), while early admixture between the two olive subspecies, as reported in Australia, may offer the possibility of new gene combinations (Besnard *et al.* 2014).

Common garden experiments with both invasive and native trees may be done to compare the phenotypes of individuals in native and invasive ranges, in order to determine the importance of genetic factors and plasticity in the expression of traits in these different environments (e.g. Kueffer *et al.* 2013; Heberling *et al.* 2016). We suggest investigating fitness and growth performance of a set of genotypes (i.e. wild, cultivated, naturalized and invasive) in different environments (i.e. native and introduced ranges). Such an experiment, however, would not be easy to carry out, and it may be difficult to obtain relevant data due to the olive's longevity. We expect that invasive olives are more likely than non-invasive olives to have traits that favour them in a changing environment; these traits include broad environmental tolerance, short juvenile periods (with rapid and profuse seedling emergence) and ability for long-distance dispersal (Hellman *et al.* 2008). Traits promoting a better adaptation to anthropogenically disturbed habitats could have also been essential to colonize abandoned pastures, and domesticated olives may have brought positively selected genetic factors (e.g. Ellstrand *et al.* 2010; Hufbauer *et al.* 2012; Thompson *et al*. 2012). Association mapping and population genetics may potentially help the identification of genomic blocks with such genes promoting adaptation to new habitats in the invasive range. For instance, selective sweeps (i.e. reduction of DNA variation in a genomic block with a mutation under recent and strong positive selection) could be observed in populations from the invasion front compared with native populations (e.g. Zenni and Hoban 2015).

Invasive olives could also provide further insight into how trees like the African olive, have successfully adapted and invaded large areas despite relatively narrow genetic variation within populations (Besnard *et al.* 2014). Inbreeding depression is expected to limit the success of introduced species, but this ‘invasion paradox’ of strong bottleneck(s) combined with invasion success has been repeatedly reported (e.g. Sakai *et al.* 2001), even in trees (Le Roux *et al.* 2008). Some authors have argued that the invasion success of genetically impoverished populations is dependent on environmental factors such as temporary or permanent release from environmental stresses in the new range (Schrieber and Lachmuth 2016). Other important factors are the initial quality of propagules and population demography (e.g. Hufbauer *et al.* 2013). In particular, further research could examine the role of domestication and successive bottlenecks in reducing the mutation load before or during the establishment of invasive olives. High genetic load is expected in large, natural populations of self-incompatible perennials, such as wild olives (Byers and Waller 1999). The European olive domestication is a complex story of inbreeding, and of admixture between distinct genetic pools (Díez *et al.* 2015). It is still unclear whether the domestication process has contributed to, or reduced the mutation load in the cultivated olive pool. For the African olive, inbreeding phases observed in the introduced range (Besnard *et al.* 2014) may have also allowed either purging or fixation of deleterious alleles. Declining heterozygosity could reduce fitness due to fixation load (e.g. Mattila *et al.* 2012), but in contrast, an efficient purging of deleterious mutations could avoid this phenomenon (e.g. Glémin 2003; Facon *et al.* 2011; Marchini *et al.* 2016). The genetic load on some traits could be compared between native (genuinely wild or cultivated) and non-native olives at different stages of the invasion process, in order to assess the impact of successive inbreeding phases (bottlenecks) in purging deleterious mutations. For each subspecies, early-growth stage performance (i.e. germination, growth) of progeny resulting from controlled crosses of native trees, of invasive trees and between native and invasive trees could be compared (e.g. Keller and Waller 2002). Such a study might help to disentangle the relative importance of the genetic load (i.e. low fixation load) and a release from stress (i.e. phenotypic plasticity) in the ‘tolerance’ of the African olive to sequential, strong bottlenecks as revealed in NSW and Hawaii (Besnard *et al.* 2014).

*Biotic interactions and monitoring of invasive populations*: The role of biotic interactions in the success of invasive olives also deserves to be investigated. The enemy release hypothesis posits that the success of some invasive species is related to the scarcity of natural enemies (e.g. parasites) in the introduced range compared with the native range (Keane and Crawley 2002). For more a decade, this hypothesis has received much attention but is probably too simplistic, because both antagonistic and mutualistic interactions can be involved with any organism which either limit or favour the spread of an exotic species.

First, interactions with microbes must be documented. Indeed, mutualistic interactions with the soil biota may facilitate plant invasions, and some invasives are known to alter soil-borne mutualists in ways that affect recipient plant communities (Richardson *et al.* 2000). Two of the strongest soil mutualisms involve mycorrhizal fungi and nitrogen fixing bacteria, both of which improve the nutrient status of their host plants (Reinhart and Callaway 2006). The interaction between these mutualists and invasives also has the potential to alter soil chemistry. An initial comparison of soil properties between native woodland areas and African olive invasion sites indicated no major differences for soil pH or key soil elements (Cuneo and Leishman 2015), however, the interaction between invasives and soil biota/chemistry deserves further investigation. The diversity of organisms associated to invasive olives and native vegetation thus needs to be studied, in order to better understand the role of mutualistic and/or antagonistic interactions in the olive invasion, which includes bacteria, mycorrhiza and microfauna such as nematodes or arthropods (Aranda *et al.* 2011; Montes-Borrego *et al.* 2014; Abdelfattah *et al.* 2015; Palomares-Rius *et al.* 2015). Co-invasion between trees and associated ectomycorrhizal fungi has been reported (e.g. Dickie *et al.* 2010); olives, however, have arbuscular mycorrhiza (Glomeromycota; Montes-Borrego *et al.* 2014) and should tend to associate with generalist, cosmopolitan fungal species or to form novel associations with native soil fungi (e.g. Nuňez and Dickie 2014). In the future, the use of techniques such as metabarcoding or metatranscriptomics (e.g. Montes-Borrego *et al.* 2014; Abdelfattah *et al.* 2015) may greatly facilitate the taxonomic and functional characterization of the microbiome and microfauna associated to the olive, both in non-invaded and in invaded woodland habitats. It may help unravel changes in local communities during olive invasion. Such changes could impact not only soil biogeochemical cycles but also affect the whole ecosystem, for example by altering competitive interactions between native and invasive plants (Callaway *et al.* 2004).

Alteration of local ecosystems through olive invasion is evident at the level of the macrofauna and invertebrates. For example, the formation of an African Olive canopy causes changes in woodland bird assemblages through changes in vegetation structure and fruit availability (NSW Scientific Committee 2010). These patterns of seed dispersal and utilization by both native and non-native animals should be quantified (e.g. Perea and Gutiérrez-Galán 2016). Furthermore, the extent of European olive cultivation throughout south-eastern Australia has highlighted the presence of several insect pests of olives. As an example, the olive lace bug (*Frogattia olivinia*; Fig. 1H), which is native to NSW and southern Queensland, is known as a pest insect of olives both in SA and NSW (Spooner-Hart *et al.* 2002; Bean 2006; P. Cuneo, personal observation). Bean (2006) found that this insect was able to impact olives through leaf damage and reduced branch growth. Unfortunately, this study was only conducted over one season and did not assess the impact of olive lace bug infestation on fruit production and long-term health of trees.

Lastly, efforts have been already made to restore habitats heavily invaded by the African olive in NSW (Cuneo and Leishman 2015). Especially, direct seeding techniques have been developed to re-establish ground layer (Fig. 1G). The impact of these practices on biotic interactions could be assessed in field experiments. The communities of microbes and microfauna (nematodes, insects) could be compared between various habitats to test the resilience of biotic interactions in restored stands. Altogether, these considerations emphasize the need to consider total ecosystem function if we want to better assess the impact of olive invasion and anticipate and control its spread.

## Concluding remarks: reclaim the past or accept a novel landscape?

Studies of woody plant invasions have shed light on many crucial aspects of invasion ecology (Richardson and Rejmánek 2011). With invasive species now well-established worldwide, the ecological role of such species in their ‘new’ habitats and the determination of appropriate ecological restoration targets (Hobbs *et al.* 2009) is now the focus of considerable debate. On a global scale, human modification of ecosystems is the major cause of biodiversity loss, a process that is being accelerated through the spread of invasive species by human activity and trade (Pimentel *et al.* 2001). Against this dominant backdrop of human ecosystem modification and invasive species impacts, some ecologists are beginning to question the feasibility of restoring ecosystems to their original or ‘historic’ condition (e.g. pre-European settlement of Australia) and whether the new combinations of species (novel landscapes) might not offer valuable ‘ecosystem services’ in a changing world (Ewel and Putz 2004).

In this review, we have described how invasive olives function as ‘ecosystem transformers’, and particularly, in the Australian context, are able to transform temperate eucalypt woodlands with a diverse grassy understory to a closed canopy system with a depauperate understory. The long life span of olive trees (100 years +) and resultant low forest floor light levels result in eventual displacement of the eucalypt woodland (including canopy trees), rather than co-existence. Olive canopy is still able to provide ecosystem services such as soil stability and fauna habitat, but this fundamental shift in vegetation structure causes changes in woodland bird and invertebrate assemblages (NSW Scientific Committee 2010; Nguyen *et al.* 2016).

Decisions about how much conservation and restoration investment is appropriate will depend on shifting cultural values about historic fidelity and ecological integrity, sentimentality about ecosystems of the past, local species diversity, priorities for livelihood and sustainability (i.e. historically faithful restorations versus ecosystem services-oriented projects), and designs for resilience (Hobbs *et al.* 2009). In the Australian context, there is an intrinsic cultural value placed on landscape identity, which is largely dominated by the eucalypt in its myriad of forms, either as a tall forest, lone paddock tree or distinctive silhouettes on a ridgeline. Understanding the biology and achieving effective control of woody invasive species such as European and African olives is about retaining ecosystem function, but also about retaining eucalypt woodlands—a core element of the Australian landscape identity.

## Sources of Funding

G.B. is supported by TULIP (ANR-10-LABX-0041) and PESTOLIVE (ARIMNet action KBBE 219262).

## Contributions by the Authors

Both authors equally contributed to this review.

## Conflict of Interest Statement

None declared.
